# Assessment of Romanian School-Aged Children’s Lifestyle and Associated Factors: A Longitudinal Study Performed Before and During COVID-19 Pandemic

**DOI:** 10.3390/children12060779

**Published:** 2025-06-14

**Authors:** Lucia Maria Lotrean, Anda Valentina Trandafir, Florina Gabor-Harosa

**Affiliations:** 1 Department of Community Medicine, Iuliu Hatieganu University of Medicine and Pharmacy, 400349 Cluj-Napoca, Romania; fgabor@elearn.umfcluj.ro; 2 Research Center in Preventive Medicine, Health Promotion and Sustainable Development, Iuliu Hatieganu University of Medicine and Pharmacy, 400349 Cluj-Napoca, Romania; trandafir.anda@umfcluj.ro

**Keywords:** children, physical activity, sleeping habits, internet use, COVID-19 pandemic

## Abstract

Background and Objectives: The COVID-19 pandemic affected children’s lifestyle, due to strict lockdown restrictions. This study evaluated Romanian children’s lifestyle prior to and during the pandemic and their associated factors. Materials and Methods: A longitudinal study was performed in 7 urban schools from Romania. Data were gathered at baseline (T1), from October to November 2019, and follow-up (T2), from December 2020 to February 2021. Results: A total of 880 children were enrolled at baseline, 484 at T2, and 350 in both evaluations. Initially, 66.3% did not perform at least one hour of physical activity per day, two thirds did not sleep at least 9 h per night during the week, and more than one third used the internet more than 2 h daily. Investigating changes before and during COVID-19 among students participating at both evaluations, a statistically significant drop in physical activity and sleep time during the week was recorded, while weekend sleep and internet use increased. Several gender- and age-related differences were noted. Physical activity and sleep patterns as well as internet use behavior at T1 predicted the same behavior at T2. Reduced sleep during the week and internet overuse were positively associated at both waves as well as longitudinally. Conclusions: These findings highlight calls for combined strategies that include parents, schools, and community, aiming to enhance a healthier lifestyle among children.

## 1. Introduction

In today’s ever more sedentary and digital times, promoting healthy movement behaviors among young people is essential for their physical health. These behaviors include daily physical activities, moderate screen time, and adequate sleep, which are important not only for immediate health benefits but also for the immune system, building healthy individuals on the long-term [1,2].

Considering these benefits, health authorities including the World Health Organization (WHO) have released specific movement behavior guidelines which recommend at least 60 min of moderate to vigorous physical activity each day, a maximum of two hours of leisure screen time, and 9–11 h of restful sleep every night for children and adolescents [1,2,3].

Children participate in several activities that compile their daily physical activity levels: commuting to school, participating in physical education classes, enjoying breaktime, teaming up in sports and dance activities, engaging in active play, and exploring surrounding playgrounds and parks [2].

Considering the array of factors that may impact lifestyle in young people, it is indispensable to examine how the COVID-19 crisis has skewed these aspects. The measures enforced by governments to control the extension of SARS-COV2 virus, including social distancing and the switch to online schooling, have led to radical changes in children’s and adolescents’ daily routines. As a consequence, teenagers are at risk because they are facing new freedoms in managing their lives, but they do not possess the ability to handle such abrupt disruptions. [4,5]. These changes in lifestyle, if persistent, could greatly increase the odds of obesity and non-communicable diseases in adulthood [6]. Important changes in eating habits and health outcomes have been reported during the pandemic, with a significant augmentation in snacking, worsening diet quality, and an increase in body mass index (BMI) [7,8,9,10,11].

Being confined at home has created for children an environment that corresponded to a prolonged summer holiday, characterized by the absence of external structure typically generated by school or extracurricular activities [8]. Data from the literature that assessed breaks from the structured school year, such as holidays and weekends, showed that changes in sleep habits and low levels of physical activity were frequent [8].

However, the call to “stay at home” that was heavily promoted during COVID-19 pandemic should not be misconstrued as an invitation to passivity. This is meaningful considering that sedentary behavior has become part and parcel of our daily living [12]. The restrictions on leisure activities during home isolation have led to increased sedentarism, mainly because of extensive time spent in the home [13,14]. Remote education, virtual meetings, and the sense of boredom resulting from limited activities have all led to the growth in screen time. [15,16,17]. However, this fact could also suggest the transition to long-lasting behavioral patterns that could persist beyond the pandemic era [8].

As children go through different stages of development, they face many challenges. The aspects of their daily lives—such as social interactions, self-perception, physical activity, schoolwork, and a pandemic crisis—play crucial roles in modelling their mental and physical well-being, including their sleep [18]. The pandemic’s lockdown measures have altered sleeping patterns in children. The tendency to sleep more observed during this time in some studies could be linked to no longer having the morning routine of a school day, permitting children to rest longer [6,17]. Moreover, inadequate sleep has led to fatigue which, in its turn, has impacted the level of physical activity carried out by children [19].

Research targeting the Romanian juvenile population and investigating their lifestyle changes during the pandemic is limited and mainly includes cross-sectional studies. Assessment of lifestyle-related behaviors and associated factors among Romanian children during several periods of time represent the first step in order to guide appropriate health promotion programs.

Therefore, our research had two objectives. The first was to assess the lifestyle–physical activity, sleep patterns, and internet use among Romanian school children before and during the COVID-19 pandemic. The second was to determine factors that influenced these components of lifestyle among children.

## 2. Materials and Methods

### 2.1. Study Design and Sample

A longitudinal study was carried out in two counties of Romania, Cluj and Alba, and included seven schools. For the implementation of the research in these schools, we received the consent of the school management. All procedures followed the guidelines of the Declaration of Helsinki. This study received approval from the Ethics Commission of “Iuliu Hațieganu” Medicine and Pharmacy University, Cluj-Napoca, Romania (134/6.05.2019 from 6.05.2019), for the entire duration of this research (May 2019–September 2021) which overlapped with the onset of the COVID-19 pandemic. No review of ethical approval was required in 2020, as the original purposes of this research were the same, including evaluation through cross-sectional and longitudinal studies of several components of lifestyle and factors which influence them among Romanian school children, but a specific objective was added as a result of the collection of the data before and during the pandemic, which was to evaluate factors influencing children’s behaviors during the COVID-19 crisis.

For recruitment of the participants, invitations were sent to 8 urban schools from two counties of Romania (Cluj and Alba). The directors were informed of the aims and the protocol of this research. A total of seven schools agreed to enroll in the survey, and one refused. Principals offered a list with available classes for participation from grades 5-8. Parents and guardians were sent letters describing the purpose of this project and were asked to provide informed consent for their children [20].

### 2.2. Procedure

This study was carried out in several phases: (1) an initial assessment (T1), (2) the implementation of educational activities promoting healthy nutrition and an active lifestyle, and (3) a follow-up evaluation (T2). Both assessments focused on children’s knowledge, attitudes, and behaviors related to lifestyle.

The baseline evaluation (T1) took place between October and November 2019 during school classes. All students from the participating classes were invited to participate. The research team distributed questionnaires to students, explaining very clearly that participation is voluntary. Children were assured of the confidentiality of their responses. They were asked to write their names on an envelope provided by the research team. After completing the questionnaire, children placed it in the envelope. The research team did not encounter any refusals, but there were some students with informed consent from their parents who were absent the day of the assessment.

With the outbreak of the COVID-19 crisis, in March 2020, all school activities moved online until the end of the school year. Therefore, the second evaluation (T2) took place from December 2020 to February 2021 and had the form of a digital survey. In Romania, the education system comprises 4 levels of education: primary school (1–4th grade), gymnasium (5th–8th grade), high school (9th–12th grade), and university. The school calendar is structured during September-June. Children who were in 8th grade in the first assessment were lost at T2.

The online questionnaire was created using Google Forms and reached target students through chat groups with the help of their teachers. Some of the items have been adapted to the context of the pandemic. Nevertheless, the main structure of the survey was similar to the baseline questionnaire to permit comparison between the first and the second assessment. After submission, the responses were collected into an Excel sheet that was downloaded by the members of the research team. Duplicate responses were excluded from the analysis [21].

Eight hundred and eighty children aged 10 to 15 years participated at T1 (mean age was 12.4 years). At T2, there were 484 students aged 12 to 15 (mean age was 13.8 years). The structure of the entire samples was not the same: there were children at T1 who dropped out at follow-up, while some students only filled in the on-line questionnaire at T2. Three hundred fifty students participated in both evaluations. At follow-up, the students who were in the VIII grade at the first assessment had already moved to high school and were not studying in the same class, so they were lost from this study, but no gender differences were noticed between those who participated at follow-up and those who dropped.

### 2.3. Instruments for Data Collection

Data were collected through a comprehensive questionnaire developed based on the literature as well as previous surveys used in various Romanian studies [22,23,24,25,26,27]. The questionnaire assessed several health risk behaviors; the questions which focused on physical activity (PA), sleep patterns, and internet use, which are the focus of this article, are depicted in Appendix A. Moreover, weight and height were measured at T1 by members of the research team.

For the present study, we analyzed children’s lifestyle changes prior to and during the COVID-19 pandemic, investigating physical activity levels, sleep habits, and internet use.

Similar with other studies, in order to be able to estimate the time of physical activity per day, the frequency and duration of involvement in physical activity in the last 7 days were assessed [26,27,28]. The first item investigated the number of days that children were involved in physical activities of different intensities (like walking, cycling, football or other ball games, running, gymnastics, etc.) in the last week. The second item referred to the time spent on PA during these days. We also paid attention to the structured sport activities, and at T1, another question inquired if the children were going to a sport club at the time when this study was performed (possibilities of answer: yes/no) [27,29]. At T2, this item was reformulated in accordance with the epidemiological conditions at that time, with children being asked if they were going to a sport club during the pandemic (possibilities of answer: yes, but the activities are suspended; yes, the activities are going on; no). Additionally, at T2, children were asked if they would have liked to do more physical activity in the previous month, as well as factors which influenced them for not doing so: fear of contagion with SARS-COV2 virus, closed sports clubs, social isolation, lack of motivation, or school closures.

On the other hand, as other studies also did, regarding sleep patterns, children were asked how many hours they sleep every night during the week and during the weekends [29,30].

Regarding internet use, similar with other studies, we evaluated the frequency and duration of internet use [26,31]. Students were asked how often they used the internet (searching for information, checking their email, visiting Facebook pages or other social platforms, and playing online games) in the last week, as well as how much time they dedicated to these activities during those days. We also asked students if they looked for topics related to a healthy lifestyle (healthy diet, physical activity, and preventing or quitting smoking) or topics related to school and socio-cultural activities in the previous month. Additionally, at T2, children responded if they had spent more than half of their day on screens (computer/TV/phone/tablet) at least once in the past month.

### 2.4. School-Based Educational Program

Before enforcing pandemic curfews, students participated in activities stimulating a healthy nutrition and an active lifestyle, previously tested within the STIVIA program among children with hearing impairments [32]. Children participated in nine educational sessions at school, with the involvement of both teachers and members of the research team. The sessions took place once every 1–3 weeks and included several topics regarding healthy nutrition, food safety, and hand hygiene, but also one of the 9 sessions was dedicated to physical activity, presenting the importance and the recommendations for an active lifestyle, as well as examples and exercises which might help and stimulate children to perform appropriate levels of PA [19,20]. The importance of sleeping was also briefly addressed together with other short messages about health, factors which influence it, and what a child can do for his own health. Each lesson contained a dedicated video. Interactive components such as games, discussions, competitions, and hands-on activities consolidated the key messages. Printed materials, including DVDs with recorded sessions, brochures, and posters, were brought in to support the teachers’ activity.

### 2.5. Statistical Analysis

Questionnaires received an identification code to ease data linkage. Before entering data, children’s names were changed with these codes.

Taking into consideration the differences in samples compositions, we performed statistical analysis for data collected at T1 and T2 among the whole study samples at each wave and separately among those followed longitudinally in both T1 and T2.

Descriptive analysis of the participants’ physical activity, sleeping patterns and internet use are presented as the prevalence (N%). We determined the mean time dedicated to PA per day and the mean time spent on the internet per day by each participant by multiplying the number of days with number of minutes per day, divided by seven.

Differences between T2 vs. T1 were examined among those who participated at both waves using the Chi^2^ test for categorical variables (less than 1 h of PA/day vs. at least one hour of PA per day; less than 9 h of sleep/night vs. at least 9 of sleep/night during week and weekends; less than 1 h vs. at least 1 h; less than 2 h vs. at least 2 h; and less than 3 h vs. at least 3 h of time dedicated to browsing the internet daily).

Factors associated with PA, sleep patterns, and internet use at both assessments were assessed using linear regressions.

At T1, the first dependent variable considered was the mean physical activity (min/day), while the independent variables included were age, gender, participating in the educational program, attendance to a sport club, BMI (Body Mass Index) which was calculated in a previous study with the formula BMI = weight (kg)/height (m)^2^ [33], children’s weight management (attempts to lose weight; attempts to gain weight), hours of sleep/night during the week, hours of sleep/night during the weekend, mean time dedicated to browsing the internet/day, and searches on the internet about healthy lifestyle in the last month. The second and the third dependent variables considered were hours of sleep per night during the week respectively during the weekend; the independent variables included were age, gender, participating to the educational program, BMI, children’s weight management, mean physical activity/day, attendance to a sport club, mean time dedicated to browsing the internet/day, whether they searched on the internet about healthy lifestyle in the last month. The fourth dependent variable included was mean time spent daily on the internet (min/day), while the independent variables included were age, gender, participating to the educational program, BMI, children’s weight management, mean physical activity/day, attendance to a sport club, hours of sleep/night during week and weekends, respectively as well as searches on the internet about healthy lifestyle in the last month.

At T2, we used the same approach, but the dependent and independent variables considered were from T2. However, BMI was excluded from the analysis because the second evaluation was conducted online, so weight and height could not be measured. Additionally, we included screen time during COVID-19 for more than half a day at least once in the last month as an independent variable.

Furthermore, we investigated the predictive factors for each behavior in the longitudinal assessment. For this analysis, we used the same procedure, but the dependent variables included were measured at T2, while the independent variables considered were measured at T1. Missing cases with regards to investigated variables were excluded only from analysis which included those variables.

More details about coded variables are provided in Appendix B.

Data analysis was performed using IBM SPSS Statistics 26 (IBM Corporation, Armonk, NY, USA). Statistical significance was considered for *p* value < 0.05.

## 3. Results

### 3.1. Children’s Lifestyle Before COVID-19 Pandemic

Baseline and follow-up data on children’s lifestyle behavior for the whole sample are presented in Table 1.

The initial results showed that 30% of children practiced daily physical activity. Two-thirds of them performed less than 60 min/day, while the recommendations of at least 60 min/day were fulfilled by 33.7% More than 30% of students attended a sport club.

Regarding sleep schedule, 66.5% of students slept less than 9 h per night during the week. On weekends, about 65% of them slept at least 9 h/night.

In terms of internet use, more than half of children used the internet daily. Approximately two-thirds of participants used the internet for 2 h/day or less, while 35.7% did so for more than two hours. Furthermore, 35.5% of participants searched online for lifestyle-related subjects, 78.6% for school-related topics, and approximately 50% for socio-cultural topics in the past month.

### 3.2. Children’s Lifestyle During COVID-19 Pandemic

At T2, in the majority of cases, children engaged in physical activity at least once a week, and 22.7% performed PA every day. Additionally, 72.9% of students performed less than 1 h of PA per day. During the COVID-19 period, 32% of children went to a sports club. In 21.5% of cases, activities were suspended at these sports clubs, and in 10.5% of cases, activities continued. Most children wished they could have engaged in more physical activity during the pandemic. Children motivated their inactivity due to fears of contagion with coronavirus, closure of sports clubs and schools, home confinement, or lack of motivation.

Taking sleep patterns into account, around 70% of students slept less than 9 h/night during the week. On the weekends, they slept more; around 73% slept at least 9 h/night, while 43% spent ≥ 10 h sleeping per night.

In 63.2% of cases, students used the internet every day, and 55.2% spent more than 2 h per day on internet. Around a third of participants stated they used the internet to look for lifestyle-related topics in the last month, 84.9% of children searched for school-related topics, and 59.3% searched for socio-cultural subjects in the last month.

More than two-thirds of children reported spending more than half of their day in front of a screen at least once in the past month.

Table 2 presents students’ lifestyle changes from T1 to follow-up among children who participated in both assessments.

Prior to and during lockdown, in the majority of cases, children performed weekly physical activity. Yet, in contrast to baseline, at follow-up, children practiced slightly less daily PA (24.0% versus 29.4%). At T2, 72.6% of them spent less than 1 h/day, versus 65.4% of children at baseline. At follow-up, rates of attending a sports clubs declined in comparison with T1. A total of 33.1% of them participated in a sport club ( and only for 10% the activities were going on), compared to 40.6% at T1. Over three-quarters of them wished to have practiced more physical activity during the pandemic but did not mainly because they worried about contracting SARS-COV-2 and closure of sports clubs and schools, followed by social isolation and lack of motivation (see Table 2).

Regarding sleep hours, 68% of children slept less than 9 h/night during workdays, contrasting with 60% at T1. On weekends, 73.7% of children slept for 9 h or more during night compared to 66.8%.

During the COVID-19 crisis, children spent more time on the internet. A total of 61.7% of them used it daily, versus 50.3% at baseline. At follow-up, more than 50% of the children used the internet over 2 h per day, as opposed to T1, when more than two-thirds of children spent two hours or less. Moreover, at T2, children searched on the internet for school-related topics more often than in T1.

Finally, 68% of children spent more than half a day on screens during the pandemic at least once in the past month.

Exploring changes from T2 versus T1, Table 2 shows that at T2 children practiced PA for at least 60 min per day less frequently than they used to at T1, a decline which was statistically significant.

Regarding sleep, during the week, there was a statistically significant drop in hours of sleep at T2 compared to T1, with a great prevalence in children sleeping <9 h/night. On weekends, however, at follow-up, children were more likely to rest for at least 9 h per night compared to baseline, a result which reached the level of statistical significance.

At T2, the percentage of children who spent ≥ 1 h, ≥ 2 h, and ≥ 3 h per day on the internet significantly increased compared to T1.

Regarding the self-perceived level of PA, children believed the level of physical activity during the pandemic was similar to the period before in 55.1% of cases. About a fifth of students stated that they had increased their physical activity, while 25.1% thought it had decreased.

### 3.3. Factors Which Influence Children’s Lifestyle

Table 3 and Table 4 present factors cross-sectionally associated with children’s lifestyle behaviors at both waves. At T1, factors correlated with increased levels of PA were male gender, attending a sports club, and an inclination to use the internet more. At T2, boys and students who participated in sports clubs or did not stay in front of the screen half a day at least once in the last month tended to perform higher levels of physical activity levels (see Table 3).

At T1, a tendency to sleep more during the week was associated with younger age, participation in the Intervention group, lower BMI, and not being interested in weight loss, as well as with a lower time spent on the internet. At T2, factors associated with increased sleep duration during the week were as follows: younger children, being a boy, no intentions of weight loss, limited internet use, and not spending more than half of the day in front of screens at least once in the last month during the pandemic period. With respect to hours of sleep during the weekend, at T1, children who had a lower body mass index, did not express intentions to lose weight, and did not attend sports clubs were more likely to sleep more. Similarly, children who did not wish to lose weight continued to sleep more during the weekend at T2 (see Table 4).

Furthermore, at T1, factors such as older age, participation in the Control group, higher BMI, not attending a sports club, being involved for more time in PA/day, and with less hours of sleep during the week were found to associate with spending more time on the internet. At T2, older children, students in the Control group, children with attempts to lose weight and sleeping less time during the week, and those who spent more than half a day in front of screens at least once in the last month during the COVID-19 pandemic were more likely to spend more time on the internet (see Table 3).

Table 5 and Table 6 describe predictive factors for children’s physical activity, sleeping patterns, and internet use. Factors that predicted an inclination towards higher PA levels at T2 were male gender, those who went to sports clubs at T1, children who were already involved in PA more time per day at T1, and those who tended to sleep more during weekdays (see Table 5).

Factors such as young age, being a boy, having a lower BMI, a higher intention to gain weight, the tendency to sleep more during the week at T1, and spending less time on the internet at T1 predicted a higher inclination towards sleeping more during the week at follow-up. Sleeping more during the weekend was anticipated by having a lower BMI at baseline, a previous T1 tendency to sleep more during weekends, and spending less time on internet/day (see Table 6).

Factors that predicted spending more time on the internet/day were older age, participation in the Control group, higher BMI at T1, attempts to lose weight, and less sleep during the week, as well as more hours of internet use at T1 (see Table 5).

## 4. Discussion

Our research showed that the COVID-19 pandemic has increased pre-existing worries about children’s inactive lifestyle [13]. Our primer results indicated that prior to pandemic, less than one in three children performed physical activity every day, and for two-thirds of them the mean time of physical activity/day did not fulfill the recommendations of at least one hour/day. These outcomes are congruent with the 2017/2018 Health Behaviour in School-Aged Children (HBSC) Survey, which reported that less than one in five teenagers followed the recommended guidelines of daily 60 min moderate-to-vigorous physical activity [34]. A study performed in 2014 among school children from urban and rural areas from the northwest part of Romania showed that only one-quarter followed the recommendations of minimum one hour of physical activity per day [26].

Moreover, baseline data showed that an important rate of children did not reach the recommended amount of sleep which is nine hours/day, during the week (around two-thirds) and on weekends (around one-third). Our initial results also showed that more than half used the internet daily and for one-third of them the average time of internet use was more than 2 h/day. This is not surprising, given the fact that children are exposed to digital devices ever earlier in their lives in this technology-dependent era. Similar results were found in research carried out in 2014 among Romanian school students which reported increased internet use, with one out of five students using internet at least 2 h daily [26]. Also, another study targeting adolescents from Romania indicated that children’s internet use fluctuated during school times and vacations. During school periods, students mostly use the internet right after school. During vacations, they spent time on the internet more evenly throughout the day. Additionally, during school time, spending three hours per day or more online was influencing the time dedicated to schoolwork [35].

At T2, the results for the entire sample of students add to the existing literature showing that during COVID-19, children have reduced physical activity but also demonstrated shifts in sleep patterns and increased screen time [17,36,37,38,39,40].

However, the literature conveys mixed findings when it comes to physical activity during the COVID-19 crisis. Several studies indicated a decline in physical activity levels among children [39,40]. A study performed during the COVID-19 period among middle school children in Cluj-Napoca showed a decrease in PA [41]. Several other studies demonstrated a reduction in organized sports activities, while regular and external activities (household chores, gardening, cycling, and walking) increased [37,42,43]. A study carried out in the northwestern part of Romania showed that amidst the pandemic, children achieved the levels of recommended PA mainly through unstructured activities. There was also a rise in screen time and unhealthy food intake [16].

In our study, the proportion of students with an average time of physical activity/day of a minimum 1 h decreased at T2, both among the whole sample as well as among the students who were followed longitudinally. Furthermore, participation in a sport club also decreased. Among students who were assessed at both waves, participation in a sport club decreased from 40% to 33% between the two waves, with only 10% having the possibility to continue attending activities. Our study also highlights changes in students’ lifestyle behavior during COVID-19 based on how they perceived it. Approximately half of them stated that their activity remained similar, while one-quarter recognized that it decreased. It is also noticed that one out of five students believed that their PA increased during the pandemic, but we did not investigate if this was because of unstructured home chore activities or indoor PA or outdoor PA (e.g., walking) performed in order to compensate for the social distance imposed by the pandemic. Nevertheless, the majority of students at T2 declared that they wished they had been practicing more PA.

In Rareş-Mihai Pop’s research, several factors were found to be affecting children’s physical activity levels during the COVID-19 pandemic, such as parental support, taking physical education classes in school, recurrent recalls on the health benefits of physical activity, and participating in extra-curricular physical activities [41]. Additionally, available data suggested that parents who endorsed physical activity and created adequate environments for such activities helped maintain children’s physical health [10,37]. In our study, children motivated their lack of physical activity through fear of SARS-CoV-2 infection, sports clubs and schools being closed, and social distancing and lack of motivation.

Our data also indicated that at T2 more than two-thirds of children slept less than 9 h/night during the week and compensated with more sleep on the weekends both among the whole sample as well as among those followed longitudinally. The pandemic impacted sleep patterns among children; although the data in the literature are mixed [8]. Some studies have reported an increase in sleep timing during COVID-19 [1,36,44], while others have observed later bedtimes and more delayed wake-up times than before the pandemic [6,45]. Several studies presented an increased in sleep duration, late bedtimes, and poor sleep quality among children and adolescents, with several differences observed based on country, period of the pandemic, age of children, and the type of sleep characteristics which were investigated [46,47,48]. In our study, we investigated only the self-perceived number of hours per night during the week and weekend, and no other characteristics were evaluated. The results show that the percentage of those who did not fulfill the recommendations of 9 h of sleep/night during the week increased at T2, but it might be noticed that children were also older by more than 1 year, so changes could be attributed at least partially to the changes in lifestyle during developmental phases.

Factors that influenced sleep problems during the pandemic were represented by limited social connections, irregular bedtimes and waking hours, absence of outdoor activities, online schooling [16], no longer commuting to school [30], and increased screen time [6]. Additionally, available data highlight that children are less active, more sedentary, and have an altered sleep schedule on days when they are not going to school [2]. As in summer vacation, the pandemic disturbed the daily routine and sleep patterns [42]. For healthy sleep patterns, children must engage in daily routines that encompass scheduled activities and reduced screen exposure [6]. Furthermore, time spent outdoors or in nature improves sleep but also encourages healthy movement behaviors, such as being more physical active and less sedentary [1,49].

Moreover, our results show that children used the internet significantly more during the pandemic. At T2, more than half of the children declared spending more than 2 h/day on the internet, and more than two-thirds revealed that during the pandemic there were days in the last month when they spent more than half the day in front of the screen both among the whole sample as well as among those who were participating at both assessments. Most of them searched the internet for school-related subjects and socio-cultural topics. Despite its benefits, information and communication technology among the young population can pose a significant threat as it may encourage sedentary behavior and an unhealthy diet as well as exposure to advertising for tobacco and alcohol products. Children may also become targets of cyberbullying [50].

The longitudinal analysis showed that, at follow-up, there were statistically significant changes in children’s lifestyle behavior. The reduction in average physical activity along with the decrease in the amount of sleep during the week were notable. On the other hand, on weekends, students tended to reach ≥9 h of sleep. Children statistically significantly increased their average time on the internet as well. These shifts could be interpreted as effects of the pandemic constraints and possibly lax parental rules. In addition, health-related behaviors in children often worsen with age.

Our study highlighted factors associated cross-sectionally with children’s lifestyle at baseline and follow-up. Younger students were more likely to sleep for more time during the week at both waves. Studies performed in other countries also showed age differences regarding sleep time during school days [51]. This finding could be attributed to the fact that young children are under more parental controls regarding bedtime, but it might be in relationship with homework requirements, planning leisure time, and being involved in several activities such as internet use. Indeed, in our study, older students spent more time on the internet, probably because they have several homework requirements but also more autonomy in terms of leisure activities. Data from other studies showed that the age difference with regard to mean time for internet use per day is subject to variations based on country, age group, and period when the study was performed [52,53]. In our sample of 11–15 years old children from urban areas of Romania before as well as during the pandemic, the use of the internet was higher among older children.

Our results concur with previous research that indicated that boys performed more physical activity than girls before the pandemic, and this tendency continued also during the pandemic [54,55]. Boys slept more at T2, probably because they had higher PA than girls.

In our study, children with a lower BMI had healthier sleeping habits during weekdays and on weekends at T1. Conversely, a higher BMI was linked to sedentary patterns like extended internet use at baseline. It was not possible to measure weight and height during the second wave, but children who did not intend to lose weight were more likely to sleep more hours both during weekdays as well as during weekends at both waves; children with attempts to lose weight were spending more time on the internet at T2. Other studies also underlined the link between appropriate sleep hygiene behaviors and healthier weight status, suggesting that families should get involved in the establishment of sleep-promoting behaviors, particularly during the school year, among children because of its benefits, including the contribution to obesity prevention and reduction [56].

Attending sports clubs was associated with greater physical activity levels at both waves. At T1, those who attended sport clubs were sleeping more hours per night during the weekdays, were less implicated in extensive sleep during the weekends, and spent less time on the internet. At T2, because of pandemic, fewer students were actively participating in sport clubs, so the protective effect disappeared. Other studies also showed that structured physical activity plays an important role in increasing PA among children and in overall child health, and a reduction increases changes in unhealthy habits, such as extensive internet usage [29]. Other studies from Romania performed among young people also underlined the protective effect of physical activity with regard to health risk behaviors [25]. The results regarding the relationship between the involvement in sport clubs and sleep time during weekdays and weekends are varying [29,57,58].

Moreover, a clear association was noticed between sleep time during the weekdays and internet use at both waves. Children who spent less time on the internet were more likely to sleep more during the night on the weekdays. At T2, spending more than half the day in front of screens during the pandemic correlated with unhealthy behaviors, such as low levels of PA, insufficient sleep during the week, and internet overuse. Other studies also showed the fact that screen time might interfere with sleep and PA [53,56].

Regarding predictive factors which had an impact on children’s lifestyle behavior, we found that male gender predicted not only increased physical activity but also increased sleep amount during the week at T2. Younger children had a tendency to sleep more during weekdays and spend less time on the internet per day. Higher BMI at baseline predicted a lower duration of sleep during weekdays and weekends and higher internet use at T2. Weight management preoccupation also predicted sleep patterns (attempts to gain weight predicted an increased sleep time during the week, while attempts to lose weight predicted higher internet use).

Engaging in a sports club at baseline forecasted higher levels of PA at T2. This might suggest that structured sports are important in keeping physical activity at great levels over time. High involvement in PA at T1 predicted increased PA at follow-up, suggesting a stability in this pattern among students who were previously more active. An increased amount of sleep during the weekdays at T1 predicted, on one hand, increased physical activity, and on the other hand, more hours of sleep during the week and reduced internet usage at T2. This finding shows the inter-relationship between an active lifestyle, good sleep patterns, and limited exposure to screens. Increased hours of sleep during weekends at baseline forecasted increased hours of sleep during weekends at T2, suggesting steady sleep patterns over time. Children with increased internet use at T1 predicted increased hours of internet use at T2.

Participation in the intervention group did not influence PA, showing that the educational program did not succeed in making a difference that lasts for more than one year between the two groups, but this might also be because of the COVID-19 pandemic lockdown and societal changes which limited several intensions and actions of children regarding involvement in PA. On the other hand, children from the control group had a higher use of the internet at T1, and this tendency remained at T2.

The results of this study should, nevertheless, be interpreted in the context of its limitations. As far as we know, although our research is among the first studies in Romania to present longitudinal data on children’s lifestyle changes prior to and during the COVID-19 pandemic, this information was self-reported. Moreover, we investigated the frequency for each lifestyle-related behavior—how many days in the last week the participants were involved in that behavior—and the duration—how many hours in those days were dedicated to that activity. This approach was used in several studies, but it gives a general overview on the behaviors, with the risk of underestimation or overestimation. According to a narrative review on methodological challenges in researching the lifestyle of school children, watching lifestyle aspects during school activities, such as physical activity during recess and sport classes, is one possible way. However, this was not an option in our study due to remote learning at follow-up. Another way of doing it is by physical activity diaries, which give detailed input but require both skills and motivation on behalf of the children for correct reporting. Several measurement instruments can verify self-reported details, such as pedometers that can unbiasedly trace the number of steps taken by children, offering a trustworthy measure of physical activity [59]. Moreover, the questionnaires were distributed during school time at T1 and online at T2, without parents involved in guiding the children in filling in the questionnaire. Second, due to challenges of the COVID-19 pandemic, as well as because the students from the last grade left the school until the second evaluation, the number of participants at T1 (880) dropped significantly at T2 (484), with only 39.7% of students participating in both evaluations. Furthermore, new participants joined at follow-up which changed the composition of the T1 and T2 samples. Third, the data were collected from urban schools from two counties of Romania during the first year of the pandemic, but the results could not be generalized to other areas or groups of students. Moreover, we did not assess the psychological effect of the pandemic on children, which could have influenced their behavior.

## 5. Conclusions

To conclude, even before the pandemic, the lifestyles of several children were already inappropriate; the COVID-19 pandemic had an impact on children’s lifestyles, with an increase in sedentary behavior, reduced levels of physical activity, inappropriate sleep patterns, and higher use of the internet, contrary to public health suggestions that people should remain active. Moreover, we assessed factors that influenced children’ lifestyle. Healthy behaviors such as regular physical activity and participation in a sport positively affected sleep and internet use. Contrariwise, unhealthy practices, such as deficient sleep and internet overuse, mutually impacted each other.

Therefore, comprehensive approaches which combine school-based education and family and community interventions, building safe and friendly environments to support and promote physical activity, are needed. Moreover, the challenges of excessive internet use by school students call for joint actions between schools and families to offer children appropriate guidance and supervision with regard to this issue, in combination with supporting appropriate sleep patterns and physical activity.

## Figures and Tables

**Table 1:** Lifestyle of the whole sample of children at T1 (N = 880) and T2 (N = 484).

	T1 N = 880 %	T2 N = 484 %
**Frequency of physical activity in the last week** 0 days 1–2 days/week 3–4 days/week 5–6 days/week Everyday	2.2 25.5 24.9 17.9 29.5	5.0 24.6 29.1 18.6 22.7
**Mean time of physical activity/day** <1 h 1–2 h >2 h	66.3 26.5 7.2	72.9 21.9 5.2
**Do you go to a sport club?** Yes No	35.7 64.3	-
**During the pandemic, are you going to a sport club**? Yes, but activities are currently suspended Yes, the activities are still going on No	-	21.5 10.5 68.0
**Did you wish to have practiced more PA in the last month, during the COVID-19 pandemic?** Yes No	-	75.4 24.6
**Why didn’t you perform PA in the last month? (multiple response)** Fear of infection with the Covid-19 virus Closed sports clubs Social isolation Lack of motivation Closed schools	-	23.3 24.6 21.9 21.5 21.3
**Hours of sleep/night during the week** 7 h or less 8 h 9 h 10 h >10 h	26.7 39.8 19.3 9.4 4.8	27.5 43.2 20.7 5.5 3.1
**Hours of sleep/night during the weekend** 7 h or less 8 h 9 h 10 h >10 h	18.1 17.2 20.2 25.5 19.0	14.3 13.1 29.3 27.4 15.9
**Frequency of using the internet/week** Less than 1 day/week 1–2 days/week 3-4 days/week 5-6 days/week Daily	6.5 10.2 13.8 10.8 58.7	5.2 8.0 9.7 13.9 63.2
**Mean time of internet use/day** <1 h 1–2 h >2 h, <3 h ≥ 3 h	38.0 26.4 21.3 14.3	27.4 17.4 24.0 31.2
**Searching for information about lifestyle on the internet during the last month** Yes No	35.5 64.5	31.6 68.4
**Searching for information related to school on the internet during the last month** Yes No	78.6 21.4	84.9 15.1
**Searching for information about socio-cultural activities on the internet during the last month** Yes No	45.5 54.5	59.3 40.7
**Screen time more than half a day at least once in the last month, during COVID-19 pandemic** Yes No	-	68.6 31.4

**Table 2:** Lifestyle before (baseline evaluation) and during the COVID-19 pandemic (follow-up) among children participating in both evaluations.

	Baseline (T1)	Follow-Up (T₂)	*p*-Value
	Total N = 350	Total N = 350	T2 vs. T1 *
**Frequency of physical activity in the last week** 0 days 1–2 days/week 3–4 days/week 5–6 days/week Everyday	1.1 25.4 22.6 21.5 29.4	3.7 22.3 31.5 18.5 24.0	
**Mean time of physical activity/day** <1 h 1–2 h >2 h	65.4 26.9 7.7	72.6 22.0 5.4	^1^ 0.041
**Do you go to a sport club?** Yes No	40.6 59.4	-	
**During the pandemic, are you going to a sport club**? Yes, but activities are currently suspended Yes, the activities are still going on No	-	23.1 10.0 66.9	
**Did you wish to have practiced more PA in the last month, during the COVID-19 pandemic?** Yes No	-	78.0 22.0	
**Why didn’t you perform PA in the last month? (multiple response)** Fear of infection with the COVID-19 virus Closed sports clubs Social isolation Lack of motivation Closed schools	-	26.0 25.7 21.7 20.6 22.0	
**BMI** Underweight Normal weight Excess weight	7.1 62.3 30.6	-	
**Children’s behavior regarding their weight** Attempts to lose weight Attempts to gain weight None	42.0 13.2 44.8	35.4 12.6 52.0	
**Hours of sleep/night during the week** 7 h or less 8 h 9 h 10 h >10 h	22.3 38.6 22.3 10.8 6.0	24.6 44.0 22.0 6.3 3.1	^2^ 0.032
**Hours of sleep/night during the weekend** 7 h or less 8 h 9 h 10 h >10 h	16.6 16.6 23.4 24.8 18.6	14.3 12.0 28.3 29.2 16.2	^2^ 0.047
**Frequency of using the internet/week** Less than one day/week 1–2 days/week 3–4 days/week 5–6 days/week Daily	7.4 14.9 15.4 12.0 50.3	4.6 7.7 11.4 14.6 61.7	
**Mean time of internet use/day** <1 h 1–2 h >2 h, <3 h ≥3 h	48.0 27.1 16.0 8.9	27.4 19.4 24.3 28.9	^3^ 0.000 ^4^ 0.000 ^5^ 0.000
**Searching for information about lifestyle on the internet during the last month** Yes No	31.4 68.6	31.1 68.9	
**Searching for information related to school on the internet during the last month** Yes No	78.6 21.4	86.3 13.7	0.007
**Searching for information about socio-cultural activities on the internet during the last month** Yes No	43.7 56.3	43.4 56.6	
**Screen time for more than half a day at least once in the last month, during COVID-19 pandemic** Yes No	-	68.0 32.0	

Note: * Differences between T_2_ vs. T_1_ were calculated with Chi^2^ test for categorical variables. ^1^ <60 vs. ≥ 60 min. ^2^ <9 h vs. ≥9 h. ^3^ <1 vs. ≥ 1 h. ^4^< 2 vs. ≥ 2 h. ^5^ <3 vs. ≥ 3 h.

**Table 3:** Factors associated with children’s PA and internet use for the entire sample of participants.

	Mean Time of PA/Day		Mean Time of Internet Use/Day	
	T1 B(CI) R^2^	T2 B(CI) R^2^	T1 B(CI) R^2^	T2 B(CI) R^2^
**Age**	B = −0.59 (−2.94; 1.75) R^2^ = 0.00	B = 1.38 (−1.39; 4.93) R^2^ = 0.00	B = 13.4 (9.54; 17.21) R^2^ = 0.05	B = 13.6 (3.72; 19.65) R^2^ = 0.02
**Gender** Male (vs. Female)	B = 11.08 (5.68; 16.41) R^2^ = 0.01	B =10.7 (3.97; 17.41) R^2^ = 0.02	B = -3.04 (−12.2; 6.17) R^2^ = 0.00	B = 8.11 (−5.57; 21.80) R^2^ = 0.00
**Participants** Intervention (vs. Control)	B = 1.99 (−3.52; 7.51) R^2^ = 0.00	B = 2.08 (−4.90; 9.07) R^2^ = 0.00	B = −21.1 (−30.3; −11.9) R^2^ = 0.02	B = −20.8 (−34.8; −6.89) R^2^ = 0.01
**Sport club** Yes (vs. No)	B = 21.4 (15.9; 26.90) R^2^ = 0.06	B =13.9 (9.08; 18.81) R^2^ = 0.06	B = −11.8 (−21.4; −2.22) R^2^ = 0.02	B = 0.58 (−9.55; 10.70) R^2^ = 0.00
**Mean time of PA/day**	-	-	B = 0.17 (0.05; 0.28) R^2^ = 0.01	B = −0.02 (−0.20; 0.16) R^2^ = 0.00
**BMI**	B = −0.31 (−0.97; 0.34) R^2^ = 0.00	-	B = 1.16 (0.05; 2.26) R^2^ = 0.02	-
**Weight management** Attempts to lose weight in the last year Yes (vs. No)	B = −1.18 (−6.74; 4.38) R^2^ = 0.00	B = 2.75 (−4.8; 9.79) R^2^ = 0.00	B = −0.69 (−10.1; 8.71) R^2^ = 0.00	B = 15.5 (1.81; 30.0) R^2^ = 0.01
**Weight management** Attempts to gain weight in the last year Yes (vs. No)	B = 2.67 (−5.19; 10.40) R^2^ = 0.00	B = −6.19 (−17.1; 4.75) R^2^ = 0.00	B = 10.1 (−3.05; 23.40) R^2^ = 0.00	B = −16.2 (−38.2; 5.79) R^2^ = 0.00
**Hours of sleep/night during the week**	B = 1.13 (−1.13; 3.62) R^2^ = 0.00	B = 2.64 (−0.80; 6.09) R^2^ = 0.00	B = −9.49 (−13.6; −5.33) R^2^ = 0.02	B = −12.5 (−19.4; −5.67) R^2^ = 0.02
**Hours of sleep/night during the weekend**	B = 0.54 (−1.14; 2.51) R^2^ = 0.00	B = −0.05 (−2.74; 2.63) R^2^ = 0.00	B = −0.29 (−3.63; 3.04) R^2^ = 0.00	B = −2.40 ( −7.81; 3.00) R^2^ = 0.00
**Mean time of internet use/day**	B = 0.06 (0.02; 0.09) R^2^ = 0.01	B = 0.01 (−0.04; 0.04) R^2^ = 0.00	-	-
**Searches on internet about healthy lifestyle** Yes (vs. No)	B = 3.59 (−2.09; 9.29) R^2^ = 0.00	B = 4.88 (−2.40; 12.11) R^2^ = 0.00	B = −6.32 (−15.9; 3.30) R^2^ = 0.00	B = −3.01 (−17.7; 11.60) R^2^ = 0.00
**Screen time half day/at least once in the last month** Yes (vs. No)	-	B = −9.03 (−16.3; −1.76) R^2^ = 0.01	-	B = 63.2 (49.6; 76.81) R^2^ = 0.14

**Table 4:** Factors associated with children’s sleeping habits for the entire sample of participants.

	Hours of Sleep/ Night During Week		Hours of Sleep/Night During Weekend	
	T1	T2	T1	T2
**Age**	B = -0.15 (−0.21; −0.09) R^2^ = 0.02	B = −0.22 (−0.32; −0.12) R^2^ = 0.05	B = 0.01 (−0.08; 0.07) R^2^= 0.00	B = −0.10 ( −0.23; 0.03) R^2^ = 0.00
**Gender** Male (vs. Female)	B = 0.09 (−0.05; 0.24) R^2^ = 0.00	B = 0.25 (0.07; 0.42) R^2^ = 0.01	B = −0.16 (−0.35; 0.01) R^2^ = 0.00	B = 0.11 (−0.11; 0.34) R^2^ = 0.00
**Participants** Intervention (vs. Control)	B = 0.23 (0.08; 0.37) R^2^ = 0.01	B = 0.09 (−0.08; 0.27) R^2^ = 0.00	B = 0.10 (−0.08; 0.28) R^2^ = 0.00	B = −0.06 (−0.29; 0.17) R^2^ = 0.00
**Sport club** Yes (vs. No)	B = 0.14 (−0.01; 0.30) R^2^ = 0.00	B = 0.10 (−0.02; 0.23) R^2^ = 0.00	B =−0.21 (−0.40; -−0.02) R^2^ = 0.01	B = 0.12 (−0.04; 0.29) R^2^ = 0.00
**Mean time of PA/day**	B = 0.001 (−0.001; 0.003) R^2^ = 0.00	B = 0.001 (−0.001;0.004) R^2^ = 0.00	B = 0.001 (−0.002; 0.003) R^2^ = 0.00	B = 0.001 (−0.001; 0.003) R^2^ = 0.00
**BMI**	B = −0.03 (−0.05; −0.01) R^2^ = 0.01	-	B = −0.02 (−0.04; −0.01) R^2^ = 0.01	-
**Weight management** Attempts to lose weight in the last year Yes (vs. No)	B = −0.25 (−0.39; −0.10) R^2^ = 0.01	B = −0.18 (−0.37; −0.01) R^2^ = 0.01	B = −0.20 (−0.39; −0.02) R^2^ = 0.01	B = −0.31 (−0.54; −0.08) R^2^ = 0.01
**Weight management** Attempts to gain weight in the last year Yes (vs. No)	B = 0.19 (−0.01; 0.40) R^2^ = 0.00	B = 0.21 (−0.07; 0.49) R^2^ = 0.00	B = 0.11 (−0.14; 0.37) R^2^ = 0.00	B = 0.17 (−0.19; 0.53) R^2^ = 0.00
**Mean time of internet use/day**	B = −0.002 (−0.003; −0.001) R^2^ = 0.02	B = −0.002 (−0.003; −0.001) R^2^ = 0.02	B = 0.001 (−0.002; 0.003) R^2^ = 0.00	B = −0.001 (−0.002; 0.001) R^2^ = 0.00
**Searches on internet about healthy lifestyle** Yes (vs. No)	B = 0.02 (−0.12; 0.17) R^2^ = 0.00	B = 0.13 (−0.05; 0.32) R^2^= 0.00	B = 0.12 (−0.06; 0.32) R^2^= 0.00	B = 0.04 (−0.19; 0.29) R^2^= 0.00
**Screen time half day/at least once in the last month** Yes (vs. No)	-	B = −0.33 (−0.52; −0.15) R^2^ = 0.02	-	B = −0.15 (−0.39; 0.08) R^2^ = 0.00

Note: BMI was calculated only at T1 based on height and weight measured by the research team, while this was not possible because the second evaluation was conducted online.

**Table 5:** Predictive factors for children’s PA and internet use among students who participated in both assessments.

	Mean Time of Physical Activity/Day	Mean Time of Internet Use/Day
	T2	T2
**Age**	B = 1.58 (−2.79; 5.96) R^2^ = 0.00	B = 17.2 (8.76; 25.70) R^2^ = 0.04
**Gender** Male (vs. Female)	B = 13.6 (5.69; 21.5) R^2^ = 0.03	B = 5.12 (−10.7; 21.0) R^2^ = 0.00
**Participants** Intervention (vs. Control)	B = 3.45 (−4.69; 11.6) R^2^ = 0.00	B = −18.9 (−34.9; −2.89) R^2^ = 0.01
**Attending a sport club at T1** Yes (vs. No)	B = 14.4 (6.45; 22.4) R^2^ = 0.03	B = 0.70 (−15.4; 16.8) R^2^ = 0.00
**Mean time of PA/day at T1**	B = 0.36 (0.27; 0.45) R^2^ = 0.15	B = 0.12 (−0.06; 0.32) R^2^ = 0.00
**BMI**	B = −0.41 (−1.40; 0.56) R^2^ = 0.00	B = 4.20 (2.30; 6.09) R^2^ = 0.05
**Weight management** Attempts to lose weight in the last year at T1 Yes (vs. No)	B = −0.66 (−8.78; 7.45) R^2^ = 0.00	B =20.3 (4.38; 36.21) R^2^ = 0.01
**Weight management** Attempts to gain weight in the last year at T1 Yes (vs. No)	B = 6.08 (−5.75; 17.9) R^2^ = 0.00	B = −3.34 CI = −26.8; 20.1 R^2^ = 0.00
**Hours of sleep/night during the week at T1**	B = 4.69 (1.15; 8.22) R^2^ = 0.01	B = −14.1 (−21.0; −7.21) R^2^ = 0.04
**Hours of sleep/night during the weekend at T1**	B = 0.30 (−2.67; 3.29) R^2^ = 0.00	B = 0.44 (−5.46; 6.35) R^2^ = 0.00
**Mean time of internet use/day**	B = −0.04 (−0.11; 0.01) R^2^ = 0.00	B = 0.45 (0.33; 0.56) R^2^ = 0.14
**Searches on internet about healthy lifestyle at T1** Yes (vs. No)	B = −1.21 (−9.85; 7.41) R^2^ = 0.00	B = −10.7 (−27.8; 6.27) R^2^ = 0.00

**Table 6:** Predictive factors for children’s sleeping patterns among students who participated in both assessments.

	Hours of Sleep/Night During Week	Hours of Sleep/Night During Weekend
	T2	T2
**Age**	B = −0.21 (−0.32; −0.10) R^2^ = 0.04	B = −0.07 (−0.21; 0.07) R^2^ = 0.00
**Gender** Male (vs. Female)	B = 0.34 (0.14; 0.55) R^2^ = 0.03	B = 0.11 (−0.15; 0.38) R^2^ = 0.00
**Participants** Intervention (vs. Control)	B = 0.14 (−0.06; 0.35) R^2^ = 0.00	B = 0.10 (−0.17; 0.37) R^2^ = 0.00
**Attending a sport club at T1** Yes (vs. No)	B = −0.05 (−2.65; 0.15) R^2^ = 0.00	B = 0.00 (−0.26; 0.27) R^2^ = 0.00
**Mean time of PA/day at T1**	B = 0.001 (−0.001; 0.004) R^2^ = 0.00	B = 0.002 (−0.002; 0.005) R^2^ = 0.00
**BMI**	B = −0.03 (−0.05; −0.01) R^2^ = 0.02	B = −0.03 (−0.07; −0.01) R^2^ = 0.01
**Weight management** Attempts to lose weight in the last year **at T1** Yes (vs. No)	B = −0.24 (−0.44; −0.03) R^2^ = 0.01	B =−0.23 (−0.50; 0.03) R^2^ = 0.00
**Weight management** Attempts to gain weight in the last year at T1 Yes (vs. No)	B = 0.42 P = (0.12; 0.73) R^2^ = 0.02	B = 0.31 P= (−0.07; 0.71) R^2^ = 0.00
**Hours of sleep/night during the week at T1**	B = 0.18 ( 0.09; 0.27) R^2^ = 0.04	B = 0.08 (−0.03; 0.20) R^2^ = 0.00
**Hours of sleep/night during the weekend at T1**	B = 0.06 (−0.01; 0.13) R^2^ = 0.00	B = 0.20 (0.10; 0.30) R^2^ = 0.04
**Mean time of internet use/day**	B = −0.003 (−0.004; −0.001) R^2^ = 0.02	B = −0.002 (−0.005; −0.001) R^2^ = 0.02
**Searches on internet about lifestyle at T1** Yes (vs. No)	B = 0.07 (−0.14; 0.29) R^2^ = 0.00	B = 0.04 (−0.24; 0.32) R^2^ = 0.00

## Data Availability

Data can be obtained from the corresponding authors.

## Appendix A

Questions related to physical activity, sleeps patterns, and internet use among children included in the questionnaire.

**Question**	**Answer**
Age	
Gender	Female/Male
1. What did you try to do about your weight in the last year?	To lose weight/To gain weight/To stay at the same weight/Nothing
2. In the last week, on how many days did you do physical activity for at least 10 min (walking, cycling, football or other ball games, running, gymnastics, etc.)?	Never/1 day/2 days/3 days/4 days/5 days/6 days/7 days
3. On days when you did physical activities, how long did you do these activities?	Half an hour/One hour/An hour and a half/2 h/More than 2 h ^a^
4. In the present, do you go to a sports club? (At the second assessment the question was changed to During the pandemic do you go to a sport club?)	No/Yes (At the second assessment the possibilities of answered were Yes, but activities are currently suspended; Yes, the activities are still going on; No)
5. Compare the activity you are doing now (last month) with the one you had before the pandemic (January-February 2020) and try to appreciate if there are changes *	It is similar/It has increased/It has decreased/I don’t know
6. In the last month, did you want to do more physical activity? *	No/Yes
7. If the answer is yes, for what reasons did you not practice more physical activity? (you can choose more than one answer) *	Fear of infection with the COVID-19 virus/Closed sports clubs/Social isolation at home/Lack of motivation/Closure of schools
8. In the present, how many hours do you sleep per night during the week?	7 h or less/8 h/9 h/10 h/More than 10 h
9. In the present, how many hours do you sleep during the night on Saturday and Sunday?	7 h or less/8 h/9 h/10 h/More than 10 h
10. How often did you access the Internet (browsing the internet for information, e-mail, Facebook or other social platforms, internet games) during the last week?	I use internet less than once a week/ 1 day a week/2 days a week/3 days a week/4 days a week/5 days a week/6 days/7 days
11. On the days when you accessed the internet, how much time did you spend on internet activities?	Less than an hour/1–2 h/3 h/4 h/ More than 4 h ^b^
12. In the past month, have you searched the internet for information about a healthy lifestyle (healthy eating or physical activity or smoking prevention and cessation)?	No/Yes
13. In the last month, did you search the Internet for information about the school that interested you?	No/Yes
14. In the last month, have you searched the Internet for information about socio-cultural or sports events/activities?	No/Yes
15. In the last month, there were days when you spend more than half of the day in front of the computer/TV/phone/tablet? *	No/Yes
*—These questions were included only in the questionnaire distributed at the second assessment during the COVID-19 pandemic. a—Hours were transformed into minutes in order to calculate the mean time/day by multiplying frequency/week with duration (minutes) and dividing by 7; for those who declared that they perform more than 2 h of PA during the days when they are involved in PA, an estimation of 150 min was used. b—Hours were transformed into minutes in order to calculate the mean time/day by multiplying frequency/week with duration (minutes) and dividing by 7; for those who declared that they stay on internet less than 1 h, a medium of 30 min was estimated, for those who stayed 1–2 h, a medium of 90 min was used, and for those who stayed more than 4 h on the internet, an estimation of 270 min was used.

## Appendix B

List of coded variables used in statistical analysis.

**Question**	**Answer**
Age	
Participants	Control = 0, Intervention = 1
BMI	Underweight” (−1), “Normal weight” (0), “Excess weight” (+1)
Weight management:	
Attempts to lose weight Attempts to gain weight	No = 0, Yes = 1 No = 0, Yes = 1
Mean time of PA/day	Min/day as calculated by multiplying frequency and duration and dividing by 7
Sport Club attendance	No = 0, Yes = 1 (T1)
Sport Club attendance during pandemic	No” (0), “Yes, but currently the activities are suspended” (1), “Yes, the activities continue” (2)
Wish to have practiced more PA in the last month, during COVID-19 pandemic?	No = 0, Yes = 1
Reasons for not performing PA: Fear of infection with the COVID-19 virus Closed sports clubs Social isolation at home Lack of motivation Closure of schools	No = 0, Yes = 1
Hours of sleep/night during the week Hours of sleep/night during the weekend	“7 h or less” (1) to “More than 10 h” (5)
Mean time for using the internet/day	Min/day calculated by multiplying frequency and duration and divided by 7
Searching on internet on topics related to lifestyle Searching on internet on topics related to school Searching on internet on topics related to socio-cultural activities	No = 0, Yes = 1
Screen time more than half a day during COVID-19	No = 0, Yes = 1

## References

[1] Moore S.A., Faulkner G., Rhodes R.E., Brussoni M., Chulak-Bozzer T., Ferguson L.J., Mitra R., O’Reilly N., Spence J.C., Vanderloo L.M. (2020). Impact of the COVID-19 Virus Outbreak on Movement and Play Behaviours of Canadian Children and Youth: A National Survey. Int. J. Behav. Nutr. Phys. Act..

[2] Guan H., Okely A.D., Aguilar-Farias N., Del Pozo Cruz B., Draper C.E., El Hamdouchi A., Florindo A.A., Jáuregui A., Katzmarzyk P.T., Kontsevaya A. (2020). Promoting Healthy Movement Behaviours among Children during the COVID-19 Pandemic. Lancet Child Adolesc. Health.

[3] Tremblay M.S., Carson V., Chaput J.-P., Connor Gorber S., Dinh T., Duggan M., Faulkner G., Gray C.E., Gruber R., Janson K. (2016). Canadian 24-Hour Movement Guidelines for Children and Youth: An Integration of Physical Activity, Sedentary Behaviour, and Sleep. Appl. Physiol. Nutr. Metab..

[4] Yang S., Guo B., Ao L., Yang C., Zhang L., Zhou J., Jia P. (2020). Obesity and Activity Patterns before and during COVID-19 Lockdown among Youths in China. Clin. Obes..

[5] Margaritis I., Houdart S., El Ouadrhiri Y., Bigard X., Vuillemin A., Duché P. (2020). How to Deal with COVID-19 Epidemic-Related Lockdown Physical Inactivity and Sedentary Increase in Youth? Adaptation of Anses’ Benchmarks. Arch. Public Health.

[6] Ventura P.S., Ortigoza A.F., Castillo Y., Bosch Z., Casals S., Girbau C., Siurana J.M., Arce A., Torres M., Herrero F.J. (2021). Children’s Health Habits and COVID-19 Lockdown in Catalonia: Implications for Obesity and Non-Communicable Diseases. Nutrients.

[7] Carroll N., Sadowski A., Laila A., Hruska V., Nixon M., Ma D., Haines J., On behalf of the Guelph Family Health Study (2020). The Impact of COVID-19 on Health Behavior, Stress, Financial and Food Security among Middle to High Income Canadian Families with Young Children. Nutrients.

[8] Burkart S., Parker H., Weaver R.G., Beets M.W., Jones A., Adams E.L., Chaput J., Armstrong B. (2022). Impact of the COVID-19 Pandemic on Elementary Schoolers’ Physical Activity, Sleep, Screen Time and Diet: A Quasi-experimental Interrupted Time Series Study. Pediatr. Obes..

[9] Jia P., Zhang L., Yu W., Yu B., Liu M., Zhang D., Yang S. (2021). Impact of COVID-19 Lockdown on Activity Patterns and Weight Status among Youths in China: The COVID-19 Impact on Lifestyle Change Survey (COINLICS). Int. J. Obes..

[10] Kenđel Jovanović G., Dragaš Zubalj N., Klobučar Majanović S., Rahelić D., Rahelić V., Vučak Lončar J., Pavičić Žeželj S. (2021). The Outcome of COVID-19 Lockdown on Changes in Body Mass Index and Lifestyle among Croatian Schoolchildren: A Cross-Sectional Study. Nutrients.

[11] Khan M.A., Moverley Smith J.E. (2020). “Covibesity”, a New Pandemic. Obes. Med..

[12] Tremblay M.S., LeBlanc A.G., Kho M.E., Saunders T.J., Larouche R., Colley R.C., Goldfield G., Gorber S. (2011). Systematic Review of Sedentary Behaviour and Health Indicators in School-Aged Children and Youth. Int. J. Behav. Nutr. Phys. Act..

[13] Rossi L., Behme N., Breuer C. (2021). Physical Activity of Children and Adolescents during the COVID-19 Pandemic—A Scoping Review. Int. J. Environ. Res. Public Health.

[14] Stockwell S., Trott M., Tully M., Shin J., Barnett Y., Butler L., McDermott D., Schuch F., Smith L. (2021). Changes in Physical Activity and Sedentary Behaviours from before to during the COVID-19 Pandemic Lockdown: A Systematic Review. BMJ Open Sport Exerc. Med..

[15] Goudeau S., Sanrey C., Stanczak A., Manstead A., Darnon C. (2021). Why Lockdown and Distance Learning during the COVID-19 Pandemic Are Likely to Increase the Social Class Achievement Gap. Nat. Hum. Behav..

[16] Nasui B.A., Ungur R.A., Nasui G.A., Popescu C.A., Hofer A.M., Dersidan S., Popa M., Silaghi H., Silaghi C.A. (2023). Adolescents’ Lifestyle Determinants in Relation to Their Nutritional Status during COVID-19 Pandemic Distance Learning in the North-Western Part of Romania—A Cross-Sectional Study. Children.

[17] Androutsos O., Perperidi M., Georgiou C., Chouliaras G. (2021). Lifestyle Changes and Determinants of Children’s and Adolescents’ Body Weight Increase during the First COVID-19 Lockdown in Greece: The COV-EAT Study. Nutrients.

[18] Cosma A., Abdrakhmanova S., Taut D., Schrijvers K., Catunda C., Schnohr C. (2023). A Focus on Adolescent Mental Health and Well-Being in Europe, Central Asia and Canada.

[19] Lin Y., Tremblay M.S., Katzmarzyk P.T., Fogelholm M., Hu G., Lambert E.V., Maher C., Maia J., Olds T., Sarmiento O.L. (2018). Temporal and Bi-Directional Associations between Sleep Duration and Physical Activity/Sedentary Time in Children: An International Comparison. Prev. Med..

[20] Trandafir A.-V., Lotrean L.M. (2024). Assessing through a longitudinal study of dietary habits among Romanian school children: Effects of COVID-19 pandemic as well as of a school based educational program for promotion of healthy nutrition. Eur. J. Nutr..

[21] Trandafir A.-V., Lotrean L.M. (2025). Education for Improving Awareness and Practices Regarding Hand Hygiene Among Romanian School Children. Sustainability.

[22] Lotrean L.M., Popa I., Florea M., Lazea C., Stanescu A.M.A., Lencu C. (2021). Actual Weight, Perceived Weight and Desired Weight of Romanian School Children by Parents and Children. Medicina.

[23] Lotrean L.M., Tutui I. (2015). Individual and Familial Factors Associated with Fruit and Vegetable Intake among 11- to 14-Year-Old Romanian School Children. Health Soc. Care Community.

[24] Lotrean L.M., Stan O., Lencu C., Laza V. (2018). Dietary Patterns, Physical Activity, Body Mass Index, Weight-Related Behaviours and Their Interrelationship among Romanian University Students-Trends from 2003 to 2016. Nutr. Hosp..

[25] Lotrean L.M., Florea M., Lencu C. (2021). Lifestyle and Cancer Prevention—Opinions and Behaviors Among Romanian University Students. Int. J. Gen. Med..

[26] Lotrean L.M., Lupșa T., del Valle M.O., Stan O., Lencu C. (2016). The use of internet and its relationship with the involvement in physical activity among Romanian school children fromurban and rural area. Palestrica Third Millenn. Civiliz. Sport.

[27] Trifescu I., Stan O., Lotrean L.M. (2017). Differences Regarding Health Risk Behaviours between Sport Club Participants and Non-Participants among Romanian High School Students. Balneo PRM Res. J..

[28] International Physical Activity Questionnaire-Short Form. https://youthrex.com/wp-content/uploads/2019/10/IPAQ-TM.pdf.

[29] Yman J., Helgadóttir B., Kjellenberg K., Nyberg G. (2023). Associations between organised sports participation, general health, stress, screen-time and sleep duration in adolescents. Acta Paediatr..

[30] Quan S.F., Combs D., Parthasarathy S. (2018). Impact of Sleep Duration and Weekend Oversleep on Body Weight and Blood Pressure in Adolescents. Southwest J. Pulm. Crit. Care.

[31] Berchtold A., Akre C., Barrense-Dias Y., Zimmermann G., Surís J.-C. (2018). Daily internet time: Towards an evidence-based recommendation?. Eur. J. Public Health.

[32] Lotrean L.M., de Vries H. (2016). Promovarea unui stil de viață sănătos în rândul elevilor: Ghid pentru cadre didactice și părinți (Promotion of a healthy lifestyle among pupils: Guide for teachers and parents.

[33] Trandafir A.-V., Fraseniuc M., Lotrean L.M. (2022). Assessment of Actual Weight, Perceived Weight and Desired Weight of Romanian School Children-Opinions and Practices of Children and Their Parents. Int. J. Environ. Res. Public Health.

[34] Europe W.H.O.R.O. (2020). Spotlight on Adolescent Health and Well-Being. Findings from the 2017/2018 Health Behaviour in School-Aged Children (HBSC) Survey in Europe and Canada. International Report. Volume 1. Key Findings.

[35] Lazea C., Popa A., Varga C. (2020). Association between Internet Use Behavior and Palpitation among Adolescents: A Cross-Sectional Study of Middle School Children from Northwest Romania. Int. J. Environ. Res. Public Health.

[36] Bates L., Zieff G., Stanford K., Moore J., Kerr Z., Hanson E., Barone Gibbs B., Kline C., Stoner L. (2020). COVID-19 Impact on Behaviors across the 24-Hour Day in Children and Adolescents: Physical Activity, Sedentary Behavior, and Sleep. Children.

[37] Kołota A., Głąbska D. (2021). Analysis of Food Habits during Pandemic in a Polish Population-Based Sample of Primary School Adolescents: Diet and Activity of Youth during COVID-19 (DAY-19) Study. Nutrients.

[38] Lopez-Iracheta R., Moreno-Galarraga L., Moreno-Villares J.M., Bueso-Asfura O.E., Martinez-Gonzalez M.A., Martin-Calvo N. (2023). The Effects of COVID-19 Lockdown on the Sleep Quality of Children. Children.

[39] Zenic N., Taiar R., Gilic B., Blazevic M., Maric D., Pojskic H., Sekulic D. (2020). Levels and Changes of Physical Activity in Adolescents during the COVID-19 Pandemic: Contextualizing Urban vs. Rural Living Environment. Appl. Sci..

[40] Wunsch K., Kienberger K., Niessner C. (2022). Changes in Physical Activity Patterns Due to the Covid-19 Pandemic: A Systematic Review and Meta-Analysis. Int. J. Environ. Res. Public Health.

[41] Pop R.-M., Grosu E.F. (2022). Physical Activity of Middle School Students from Cluj-Napoca during the Covid-19 Pandemic. Rev. Romaneasca Pentr..

[42] Schmidt S.C.E., Anedda B., Burchartz A., Eichsteller A., Kolb S., Nigg C., Niessner C., Oriwol D., Worth A., Woll A. (2020). Physical Activity and Screen Time of Children and Adolescents before and during the COVID-19 Lockdown in Germany: A Natural Experiment. Sci. Rep..

[43] Schnaiderman D., Bailac M., Borak L., Comar H., Eisner A., Ferrari A., Giannini G., Risso F., Vetere C., Garibotti G. (2021). Psychological Impact of COVID-19 Lockdown in Children and Adolescents from San Carlos de Bariloche, Argentina: Parents’ Perspective. Arch. Argent. Pediat..

[44] Pietrobelli A., Pecoraro L., Ferruzzi A., Heo M., Faith M., Zoller T., Antoniazzi F., Piacentini G., Fearnbach S.N., Heymsfield S.B. (2020). Effects of COVID-19 Lockdown on Lifestyle Behaviors in Children with Obesity Living in Verona, Italy: A Longitudinal Study. Obesity.

[45] Liu Z., Tang H., Jin Q., Wang G., Yang Z., Chen H., Yan H., Rao W., Owens J. (2021). Sleep of Preschoolers during the Coronavirus Disease 2019 (COVID-19) Outbreak. J. Sleep Res..

[46] Richter S.A., Ferraz-Rodrigues C., Schilling L.B., Camargo N.F., Nunes M.L. (2023). Effects of the COVID-19 pandemic on sleep quality in children and adolescents: A systematic review and meta-analysis. J. Sleep Res..

[47] Zhou S.-J., Zhang L.-G., Wang L.-L., Guo Z.-C., Wang J.-Q., Chen J.-C., Liu M., Chen X., Chen J.-X. (2020). Prevalence and Socio-Demographic Correlates of Psychological Health Problems in Chinese Adolescents during the Outbreak of COVID-19. Eur. Child Adolesc. Psychiatry.

[48] Weaver R.G., Beets M.W., Perry M., Hunt E., Brazendale K., Decker L., Turner-McGrievy G., Pate R., Youngstedt S.D., Saelens B.E. (2019). Changes in Children’s Sleep and Physical Activity during a 1-Week versus a 3-Week Break from School: A Natural Experiment. Sleep.

[49] Tremblay M., Gray C., Babcock S., Barnes J., Bradstreet C., Carr D., Chabot G., Choquette L., Chorney D., Collyer C. (2015). Position Statement on Active Outdoor Play. Int. J. Environ. Res. Public Health.

[50] Lotrean L.M., Ailioaiei R., Stan O. (2016). Use of Information Technology by Adolescents and Young People and Its Effect on Health Promotion. Balneo PRM Res. J..

[51] Ohayon M.M., Carskadon M.A., Guilleminault C., Vitiello M.V. (2004). Meta-analysis of quantitative sleep parameters from childhood to old age in healthy individuals: Developing normative sleep values across the human lifespan. Sleep.

[52] Mori T., Aoki T., Oishi K., Harada T., Tanaka C., Tanaka S., Tanaka H., Fukuda K., Kamikawa Y., Tsuji N. (2024). Relative age effect on the physical activity and sedentary behavior in children and adolescents aged 10 to 18 years old: A cross-sectional study in Japan. BMC Public Health.

[53] Parenting Children in the Age of Screens. https://www.pewresearch.org/internet/2020/07/28/childrens-engagement-with-digital-devices-screen-time/.

[54] Telford R.M., Telford R.D., Olive L.S., Cochrane T., Davey R. (2016). Why Are Girls Less Physically Active than Boys? Findings from the LOOK Longitudinal Study. PLoS ONE.

[55] Sánchez-Sánchez E., Ramírez-Vargas G., Avellaneda-López Y., Orellana-Pecino J.I., García-Marín E., Díaz-Jimenez J. (2020). Eating Habits and Physical Activity of the Spanish Population during the COVID-19 Pandemic Period. Nutrients.

[56] Moreno J.P., Wood A.C., Reichek B., Dadabhoy H., Baranowski T., Thompson D., O’Connor T.M. (2023). Examination of parent-reported differences in children’s daily screen use, sleep, and sleep hygiene behaviors during the school year and summer and their association with BMI. Sleep Health.

[57] Saevarsson E.S., Rognvaldsdottir V., Stefansdottir R., Johannsson E. (2021). Organized Sport Participation, Physical Activity, Sleep and Screen Time in 16-Year-Old Adolescents. Int. J. Environ. Res. Public Health.

[58] Cavadini C., Decarli B., Grin J., Narring F., Michaud P.-A. (2000). Food Habits and Sport Activity during Adolescence: Differences between Athletic and Non-Athletic Teenagers in Switzerland. Eur. J. Clin. Nutr..

[59] Lotrean L.M., Popa M., Santillan E.A., Florea M. (2014). Methodological challenges in research regarding the lifestyle of school children. Rev. Cercet. Si Interv. Soc..

